# 532 nm Low-Power Laser Irradiation Recovers γ-Secretase Inhibitor-Mediated Cell Growth Suppression and Promotes Cell Proliferation via Akt Signaling

**DOI:** 10.1371/journal.pone.0070737

**Published:** 2013-08-07

**Authors:** Yumi Fukuzaki, Haruna Sugawara, Banri Yamanoha, Shinichi Kogure

**Affiliations:** 1 Department of Bioinformatics, Graduate School of Engineering, Soka University, Hachioji, Tokyo, Japan; 2 Department of Environmental Engineering for Symbiosis, Faculty of Engineering, Soka University, Hachioji, Tokyo, Japan; MGH, MMS, United States of America

## Abstract

**Background and Objective:**

The γ-secretase inhibitor (GSI) has been shown to inhibit expression of amyloid beta (Aβ), but GSI also has a side effect of reducing cell survival. Since low-power laser irradiation (LLI) has been known to promote cell survival, we examined whether 532 nm LLI can rescue the GSI side effect or not.

**Study Design/Materials and Methods:**

The human-derived glioblastoma cells (A-172) were cultured in 35 mm culture dishes or 96-well plate. The center of dish or selected wells was irradiated with 532 nm laser (Nd:YVO4, CW, 60 mW) for 20, 40 and 60 min, respectively. The irradiated cells were photographed at immediately after, 24 and 48 h later and counted. GSI was supplemented in medium 3 h before LLI. The MTT assay was also used to estimate viable cells at 48 h after irradiation. The expression of phosphorylated Akt (p-Akt) or phosphorylated PTEN (p-PTEN) was examined by immunofluorescent staining and measured by fluorescence intensity using the software (BZ-9000, KEYENCE, Japan).

**Results:**

GSI application depressed cell proliferation as well as cell survival compared to control. GSI down-regulated Aβ but up-regulated p-PTEN and suppressed p-Akt. Application of 532 nm LLI in the presence of GSI significantly recovered the GSI-mediated effects, i.e., LLI could decrease elevated p-PTEN, while increased p-Akt expression with keeping Aβ suppression. The LLI effects had a dose-dependency.

**Conclusion:**

We confirmed that GSI potently suppressed intracellular Aβ and decreased cell survival. We conclude that a combination of GSI application and 532 nm LLI can increase cell proliferation via Akt activation while keeping PTEN and Aβ suppressed.

## Introduction

Alzheimer's disease is a serious problem for aged individuals. The number of patients will almost double every 20 years, reaching 65.7 million in 2030 and 115.4 million in 2050 [1]. Amyloid beta (Aβ) is considered as a pathogenic agent of Alzheimer’s disease that is processed from amyloid precursor protein (APP) by γ-secretase (GS) [2]. Intracellular as well as extracellular accumulations of Aβ result in nerve cell toxicity [3]. As GS activity is essential for the release of intact Aβ, γ-secretase inhibitors (GSIs) have been contemplated for the treatment of Alzheimer’s disease. Since GSIs have been shown to decrease Aβ production after administration to transgenic mice overexpressing human APP [4], they were considered as useful drugs to lower Aβ accumulation for long-term treatment in human patients [5], [6].

Despite of the potential benefit, GSIs could have heavy side effects. It is well known that GS-mediated intracellular processes activate Notch signaling pathway, which is associated with cell proliferation and differentiation [7]–[9]. Notch regulates the expression of a phosphatase PTEN (Phosphatase and Tensin homolog deleted from chromosome) via intracellular Notch (ICN), the intracellular moiety of Notch. ICN, when released from Notch by GS, suppresses the expression of PTEN, which dephosphorylates a phosphoinositides that is critical for activation of Akt, a Ser/Thr kinase [10]. Activated Akt plays key roles in mediating cell proliferation, cell survival (anti-apoptotic), cell-cycle progression, differentiation, transcription, translation, and glucose metabolism [11], [12]. Therefore, although GSIs could be effective for treating Alzheimer’s disease with their inhibitory role of Aβ expression and accumulation, they have unwanted side effects of suppressing cell proliferation and survival by inhibiting Akt activation via PTEN elevation. These dual aspects of GSIs await other novel drugs or treatments that ameliorate the side effects.

It has been reported that the low-power laser irradiation (LLI) can promote cell proliferation and survival. Mester et al. first reported such effects on intractable skin ulcer in 1968 [13]. Since then, there are many studies showing LLI-mediated cell proliferation and survival in various fields including wound healing, reumatoid arthritis, tendinopathy, osteoarthritis [14]–[18]. In studies using cell culture systems, it was demonstrated that 532 nm LLI promoted proliferation of B-14 (Chinese hamster ovarian cell line) cells without inducing cell death [19]. Another study showed the 532 nm LLI on blood platelets can trigger signal transduction, leading to platelet activation, as well as the gradual loss of natural platelet reactivity and platelets' ability to respond to activating agents [20]. Mechanisms of these cell proliferating effects of 532 nm LLI are unclear, but recent work using 632.8 nm LLI indicated that Akt activation is involved in prevention of cell apoptosis [21].

Here we examined the effects of 532 nm LLI on cell proliferation in human-derived glioblastoma (A-172) and aimed to reveal mechanisms underlying LLI effects by investigating the involvement of the Notch-Akt signaling pathway.

## Materials and Methods

We performed all experiments in accordance with the Declaration of Helsinki and Guide for Animal Experimentation at Soka University.

### Laser Irradiation Method

A diode laser apparatus (Nd:YVO_4_, CW, 532 nm, 0–180 mW) was used. The experiment was conducted in a clean bench under 37°C and 5% CO_2_. The laser beam was reflected on a mirror and introduced to cells from the top to the bottom. The averaged power was 60 mW and the irradiated area was 7.1 mm^2^, thus the power density was 845 mW/cm^2^. In experimental group, the center of dish or well was irradiated for 20, 40 and 60 min with an energy density of 10.1, 20.3, 30.4×10^2^ J/cm^2^, respectively.

### Cell Culture of Glioblastoma (A-172)

The human-derived glioblastoma A-172 cell line was obtained from JCRB (#0228). The cells were cultured in 25 cm^2^ flasks with DMEM containing 10% fetal bovine serum and incubated in an atmosphere of 5% CO_2_, 95% air at 37°C. The medium was changed once every three days to maintain cell growth. Trypsinization was performed using 0.1% trypsin-PBS once the cells reached confluency (10^6^ cells). Cells were plated in 35 mm dish, 96-well plates (MS-8096F, Sumitomo Bakelite Co. Ltd., Tokyo, Japan), or LAB-TEK Chamber Slide (Nalge Nunc International, New York, USA) with 10% FBS-DMEM medium at 6×10^3^ cells/ml.

### Cell Counting by Microscopic Observations

On the day following plating, culture wells on the clean bench maintained at 37°C and in an atmosphere containing 5% CO_2_ were photographed with a digital camera before LLI. After taking photographs, the plate was returned to the incubator. Photos were taken immediately after LLI, and 24 and 48 h after LLI. The photos were displayed on a PC monitor and cells were counted per unit area in all areas including the directly irradiated area.

### Cell Viability by MTT Assay

Cell viability was measured by the MTT (3-(4,5-dimethylthiazol-2-yl)-2,5-diphenyl-2H- tetrazolium bromide) colorimetric assay. The A-172 cells were plated at 0.3 × 10^4^ cells/well in a 96-well plate (200 µl/well) and incubated for 12 hours. At 48 h after LLI, the culture medium was removed, and 200 µl of fresh medium containing 20 µl of MTT (6 mg/ml; Sigma-Aldrich Japan, Tokyo, Japan) was added to each well. The cells were incubated at 37°C for 3 h. Colored precipitates were extracted with 100 µl of acidic isopropanol at room temperature for 1 h, and the absorbance was measured at 570 nm using a plate reader.

### Immunofluorescent Staining Method

The cells were rinsed three times with PBS after LLI. They were fixed with 4% PFA, treated with 0.1% Triton X-100 and blocked by 1% BSA-PBS. Primary antibody against phospho-Thr308-Akt (1∶800, Cell Signaling), phospo-Ser473-Akt (1∶800, Promega), phosphor-Ser380/Thr382/383-PTEN (1∶100, Cell Signaling), or Aβ-Amyloid (1∶50, Cell Signaling) was added and left overnight at 4°C. Next day, secondary antibody (Rhodamine, FITC) was added and left for 3 h under room temperature. After washing with PBS, glycerol was added and cover glass was put on. Stained cells were observed by a fluorescent microscope (BZ-9000, KEYENCE, Tokyo, Japan). The center of each well was photographed with same exposure time using a 20x objective lens after focusing on cells under phase contrast. The maximum intensity in irradiated area was measured using an optional software (BZ-analysis application, KEYENCE) without applying any image enhancement. However, the haze reduction was used to make fluorescent images clearer.

### Pretreatment Cells with γ-secretase Inhibitor (GSI)

DAPT, a typical agent of GSIs, was cryopreserved at 1 mM in DMSO. DAPT was added directly to FBS-DMEM medium at 25 µM and applied to cells 3 h before LLI. DMSO (1% in the medium) showed harmless effects in preliminary experiments (data not shown).

### Analysis of ATP Level in Cell Lysate

An ATP/ADP ratio assay kit (EnzyLightTM, BioAssay Systems, CA, USA) was used to quantify ATP amount. A-172 cells were plated at 0.5×10^4^ cells/well in a 96-well plate (200 µl/well) and incubated for 12 hours. The culture medium was removed after LLI. 90 µl ATP reagent was added to each 96-well and mixed by tapping the plate. After 1 min, luminescence (RLU A) was read on a luminometer (ATTO BIO-INSTRUMENT, Tokyo, Japan). 10 minutes after measuring RLU A, the luminescence of samples was read again (RLU B). This measurement provides the background prior to measuring ADP. Immediately following RLU B measurement, 5 µl ADP reagent was added to each well and mixed by pipetting up and down. After 1 min, luminescence (RLU C) was read for ADP level. The following formula was used to calculate ATP/ADP ratio: (RLU C – RLU B)/RLU A.

### Statistical Analysis

All values were presented as mean ± SD. Student’s two-tailed non-paired t-test and one-way ANOVA were used to analyze statistical differences between 2 groups or among multiple groups, respectively.

## Results

### Effects of 532 nm LLI on the Number of A-172 Cells

**Figure 1:**
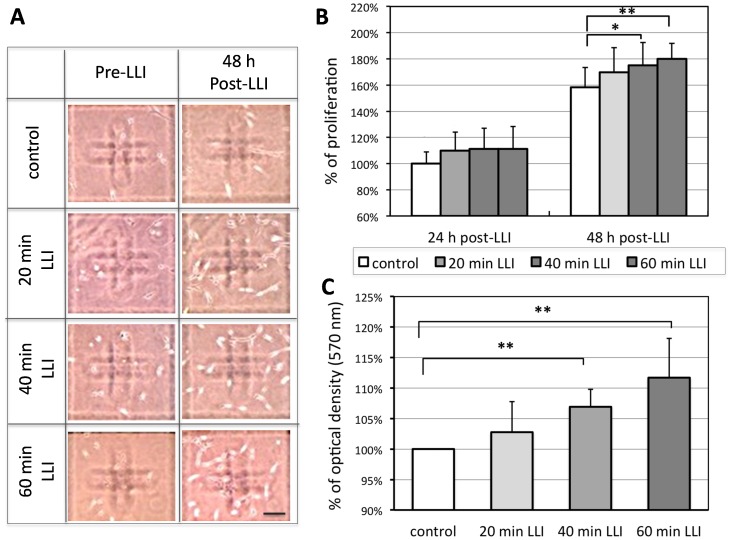
Effects of LLI on the number of A-172 cells. A: Sample images of A-172 cells under light microscope. The number of cells increased 48 h post-LLI (right column) after 20, 40, 60 min LLI compared to pre-LLI (left column). Cal.: 100 µm. B: Proliferation ratio (the ratio of cell number at 24 or 48 hours following LLI and cell number before LLI) was normalized to control (no LLI) (n = 12 for each group). C: A summary of colorimetric analysis by MTT staining performed at 48 h after LLI (each group: n = 16). The optical density of each group was normalized to the value of control group (no LLI) at 48 h after initial condition. Asterisks: one-way ANOVA, * p<0.05, ** p<0.01.

We have examined if LLI affects the proliferation of A-172 cells grown in culture. The cells were plated at 0.3 × 10^4^ cells/well in 96-well plates and were irradiated with 532 nm LLI for 0 (no LLI: control), 20, 40, and 60 min. The number of cells remaining at 24 and 48 hours after LLI was counted and compared to that before LLI (Pre-LLI) (Fig. 1). At 24 h after LLI, the proliferation ratio, the number of remaining cells after LLI normalized to that of untreated control, was 110±14%, 111±16%, or 111±17% for 20 min, 40 min, or 60 min LLI, respectively (Fig. 1B-left bars, no significances among any groups). At 48 h, they were 158±15% for control, 170±19%, 175±18%, or 180±12% for 20 min, 40 min, and 60 min LLI, respectively (Fig. 1B, right bars, n = 12, p<0.05 for 20 and 40 min LLI, p<0.01 for 60 min LLI).

In addition to cell counts, we also used a calorimetric method to quantify the proliferation and survival in cell culture using the MTT assay. The mean optical density at 570 nm at 48 hours after 40 or 60 min LLI increased significantly over non-irradiated control (Fig. 1C, n = 16, p<0.01 for 40 and 60 min LLI), while little change was observed with 20 min LLI.

### Expressions of p-Akt and p-PTEN by 532 nm LLI

**Figure 2:**
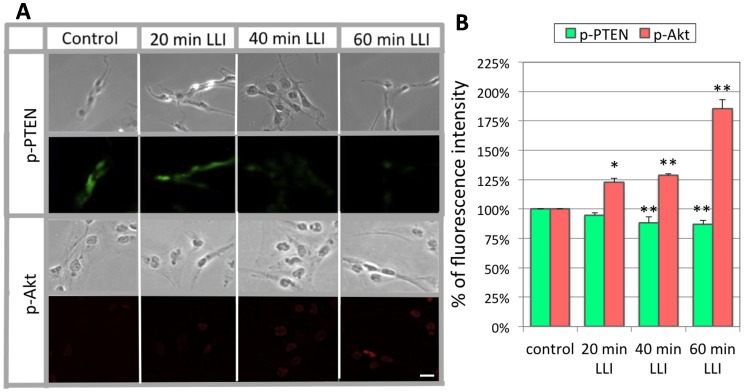
Immunofluorescent staining of Akt and PTEN. A: The LLI effects on immunofluorescence staining of p-PTEN and p-Akt in cultures cells (bottom). Corresponding phase contrast micrographs are shown on top of each fluorescent image. It should be noted that tumor cells normally proliferate in high proportions and p-Akt is often highly expressed in cancer cells of different natures, whereas our result of control group shows low level of p-Akt expression due to haze reduction. Cal.: 100 µm. B: Average fluorescence intensity for p-PTEN or p-Akt normalized to the control value. Asterisks: one-way ANOVA, * p<0.05, ** p<0.01.

To examine possible involvement of Akt signaling in the LLI-mediated proliferation, we investigated Akt as well as PTEN activation (Fig. 2). Anti-phosphorylated Akt (p-Akt) antibodies detect activated (phosphorylated) Akt molecules, while anti-phosphorylated PTEN (p-PTEN) antibodies detect PTENs that removed the phosphate group from activated phosphoinositides and retained it [22]. With 20 min LLI, the intensity of p-PTEN immunofluorescence did not change significantly (95±2% of control). However, with 40 and 60 min LLI, the p-PTEN immunofluorescence intensity decreased significantly over control (88±5%, p<0.05, for 40 min; 87±3%, p<0.05, for 60 min) (n = 120∶4 experiments and 30 selected cells each). In contrast, p-Akt immunofluorescence intensity significantly increased (122±4%, 129±1%, and 185±8% for 20, 40, and 60 min LLI over control, p<0.05 or p<0.01).

### Effects of a Combined Application of GSI and LLI on Cell Proliferation

**Figure 3:**
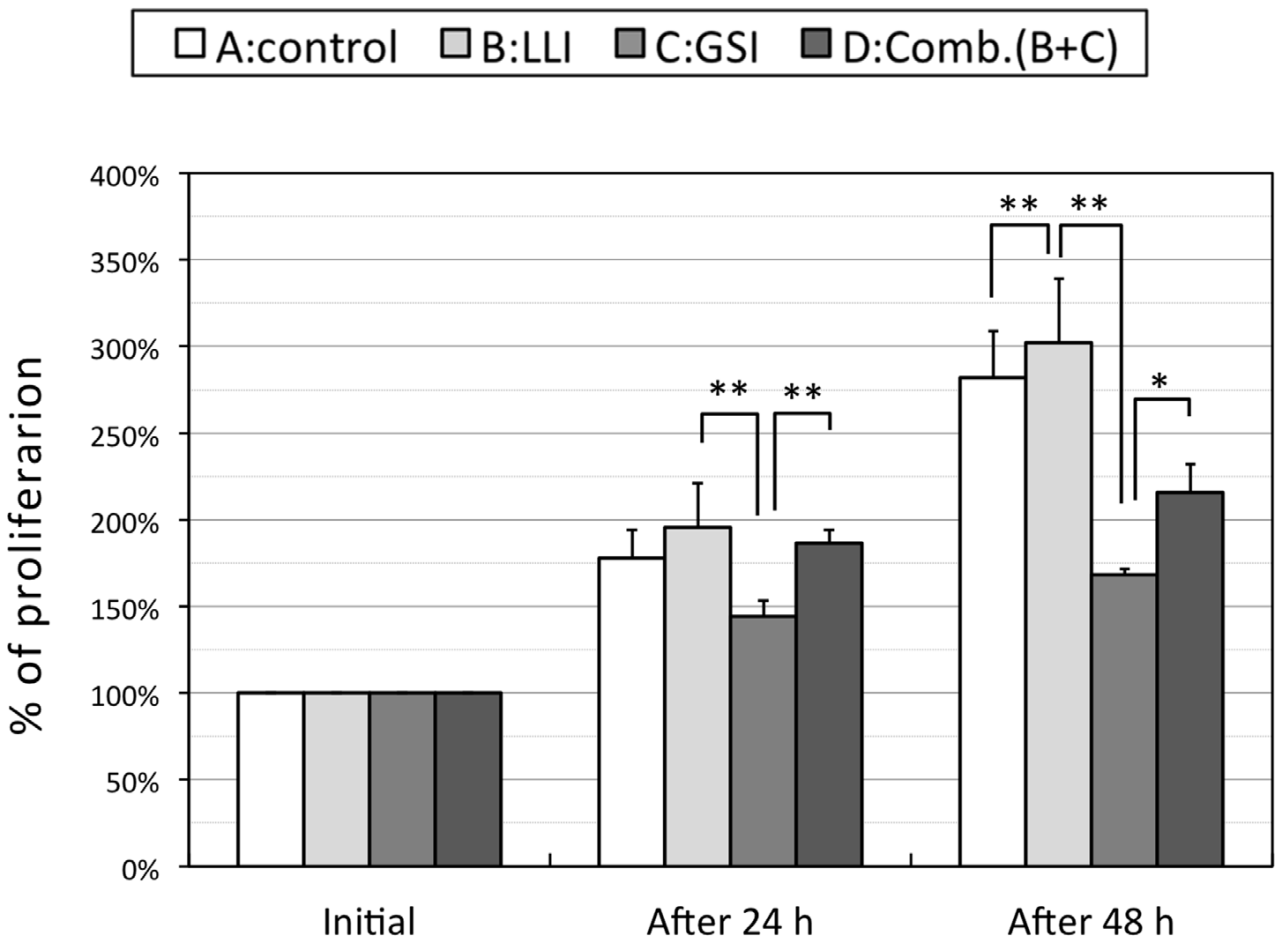
Effects of LLI, DAPT and combined application of both on cell proliferation. Proliferation ratios for each group at 24 h and 48 h after LLI for control (white bars), 60 min LLI (light grey bars), DAPT (grey bars) or the combination of LLI and DAPT (dark grey bars) are shown. Asterisks: one-way ANOVA, * p<0.05, ** p<0.01.

DAPT, a typical agent of GSIs, has been previously shown to retard cell proliferation by reducing generation of ICN [23]. We also tested this agent in A-172 cells in 13 experiments. At 24 h after LLI, the standardized proliferation ratios were 178±16% for control group, 196±26% for experimental group which received 60 min LLI only, 144±9% for pretreated group with GSI, and 186±8% for group received both applications, respectively. At 48 h after LLI, they were 282±27, 302±37, 168±4, and 216±16%, respectively (Fig. 3). There were statistical significances between some pairs of groups as shown in Fig. 3 (p<0.01 or p<0.05). It is worthy of note that the LLI was able to rescue the DAPT-induced reduction of cells by approximately (186–144 = ) 42% at 24 h and (216–168 = ) 48% at 48 h after LLI.

### Effects of LLI on Intracellular Signaling Molecules under GSI

**Figure 4:**
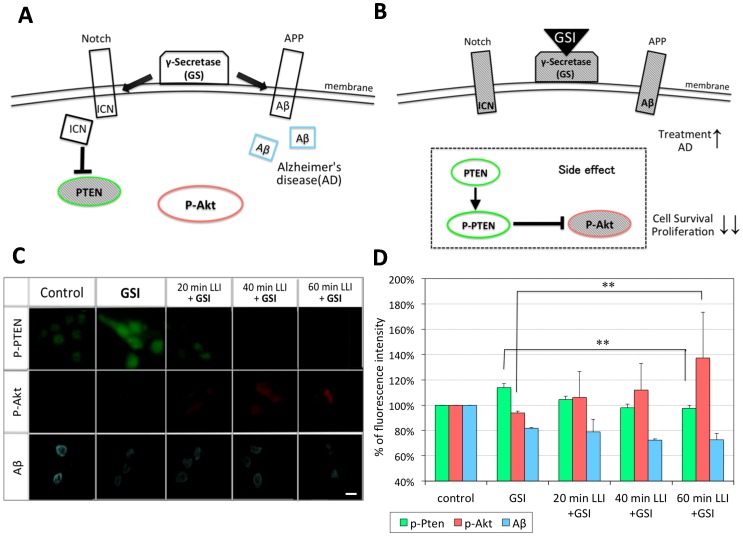
Effects of LLI on the fluorescence intensities of p-Akt, p-PTEN and Aβ. A, B: Schematic diagrams of Notch and APP signaling pathways. GS can cleave APP and Notch, making Aβ and ICN, respectively (A). GSI inhibits Aβ expression but also inhibits ICN expression (B), a side-effect against cell survival through PTEN activation (dashed box in B). C: LLI effects on immunofluorescence staining of p-Akt, p-PTEN and Aβ in cells pretreated with GSI. Cal.: 100 µm. D: Average fluorescence intensity for p-PTEN (green bars), p-Akt (pink bars) and Aβ (blue bars) was normalized to control. Asterisks: one-way ANOVA, ** p<0.01.

Fig. 4A describes the action of GS in relation to Notch and APP processing. GS cleaves Notch as well as APP at plasma membrane, making ICN and Aβ, respectively. When GS is inhibited by GSI, ICN level will be reduced and PTEN expression will increase, resulting in suppression of Akt signaling pathway and in inhibition of cell proliferation and survival (Fig.4B). Similarly, Aβ level will decrease as more uncleaved APP will remain.

We next tested if the LLI-mediated rescue of GSI-induced reduction in cell number (Fig. 3) involves activation of Akt and the reduction of PTEN. As described above, A-172 cells were treated with DAPT for 3 hours, exposed to LLI for 20, 40, and 60 min, and fixed at 15 min for the immunofluorescent staining of p-Akt and p-PTEN (Fig. 4C). Immunofluorescence intensity was quantified for each protein and normalized to non-DAPT treated control (Fig. 4D) (n = 120∶4 experiments and 30 selected cells each). As expected, DAPT treatment increased the expression of p-PTEN (114±3% when standardizing untreated control as 100%, p<0.01), suggesting that PTEN expression has increased due to inhibition of GS and reduction of ICN. We also examined if soluble Aβ is reduced by immunofluorescent staining using anti-Aβ antibodies. As would be expected for the inhibition of GS, Aβ was reduced in DAPT-treated cells (82±1%, p<0.01). We then tested if LLI could alter the expression of p-PTEN as well as Aβ. Simultaneous application of 20 min LLI reduced p-PTEN to the control level, but did not affect already reduced Aβ level. Effects of 40 and 60 min LLI were similar to 20 min LLI. These data suggest that LLI, within 20 min, acts specifically on the Notch pathway to reduce PTEN expression, but does little to the APP processing and homeostasis of Aβ.

Immunofluorescence intensity of p-Akt was reduced slightly from the control level in DAPT-treated cells (94±1%, p<0.01). This decrease was rescued by LLI. With 20 min LLI, the immnofluorescent staining of p-Akt was recovered to the control level. With 40 and 60 min LLI, the intensity significantly increased 112±21% (p<0.01) and 137±36% (p<0.01) above the control level, respectively. These data suggest that LLI could recover DAPT-induced reduction of p-Akt in a dose-dependent manner.

### Effects of LLI on ATP Level in Cell Lysates

**Figure 5:**
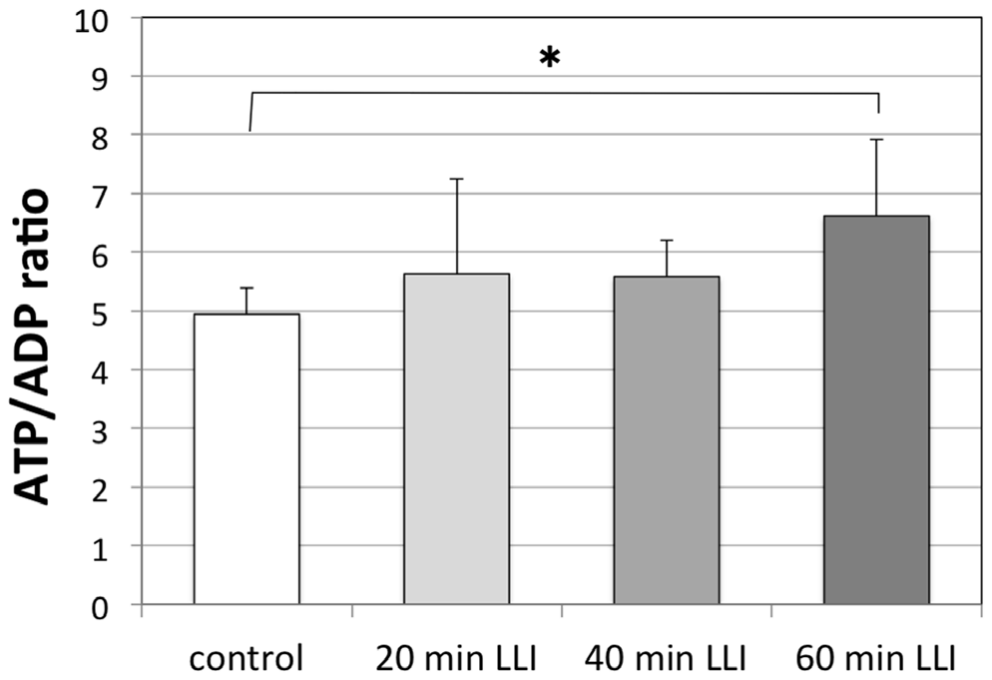
Effects of LLI on ATP level in cell lysates. The ATP/ADP ratio measured using a luminescence-based assay for control and for 20, 40 and 60 min LLI. The luminescent densities for ATP and ADP were measured 48 h after LLI treatment. The ATP/ADP ratio was calculated and averaged (n = 5). Asterisks: one-way ANOVA, * p<0.05.

To test the possibility that LLI-induced cell proliferation involves ATP elevation, we examined ATP level in cell lysates using ATP/ADP ratio assay kit (Fig. 5). Mean luminescence after 60 min LLI increased significantly over that of control group (p<0.05, n = 5), while little changes were observed for 20 or 40 min LLI groups.

## Discussion

In the present study, using conventional cell counting and the colorimetric MTT assay, we demonstrate that 532 nm LLI (60 mW) significantly increased viability of human-derived glioblastoma A-172 cells. The effect showed a LLI dose-dependency with 60 min LLI being the most effective for cell proliferation. These findings contrast to studies using LLI of other wavelengths in the same cell type. The infra-red 808 nm LLI delayed cell cycle and suppressed cell proliferation [24], and the 405 nm LLI promoted the cell death in A-172 cells [25]. Thus, the effect of LLI on the cell proliferation appears to depend on the wavelength. Indeed, LLI wavelength-dependent effects were previously reported in various cell types [26].

Different effects of LLI wavelength on cell proliferation and survival may depend on photoacceptors present in mitochondria, especially those in the mitochondrial respiratory chain [27]. Primary photoacceptors include cytochromes c oxidase (red and near-infrared light region), NADH-dehydrogenase (blue spectral region), and cytochromes b, c1 and c (green light region). Their cofactors are porphyrins (for cytochrome c oxidase and cytochromes b, c1 and c), or flavins (for NADH-dehydrogenase), respectively [28]. These photoreceptors and cofactors contribute to the generation of ATP, a critical source of chemical energy in cells. Mitochondria are the center of many diverse cellular functions such as signaling, cellular differentiation, cell death, as well as the control of the cell cycle and cell growth [29]. Thus photoacceptors in mitochondria upon light absorption may regulate the level of ATP to support cell survival. However, considering that LLI has different biological effects on different cells, it is difficult to conceive that the regulation of mitochondrial photoacceptors is the only mechanism underlying LLI-mediated cell proliferation. It is likely that final consequences of specific photobiological effects are determined not at the level of primary reactions in the mitochondrial respiratory chain but based on secondary cellular signaling [27]. It will be important to characterize how proteins and enzymes expressed in each cell type contribute to photobiological effects.

The present study focused on the effect of LLI on the Notch-PTEN-Akt pathway in glioblastoma. We showed a reciprocal expression of p-PTEN inactivation and p-Akt activation in Fig. 2. Previous work of others showed the effects of LLI on the mitogen-activated protein kinase (MAPK) pathway [30] and the ROS-Src pathway [31]. To our knowledge, this is the first to report that LLI at any wavelength influenced the Notch pathway. Previously, it was suggested that Akt plays a key role for cell survival and proliferation [32]. Using FRET, it was demonstrated that 632 nm LLI increased cell proliferation via the activation of Akt signaling pathway [33]. It was also reported that 632.8 nm LLI inhibits the expression of p21 [34], a molecule that arrests the cell-cycle, and enhances the cell-cycle progress via its phosphorylation by Akt [35]. Recent investigations implicate an important role of Akt in mitochondria for the regulation of cell growth. The expression level of Akt in mitochondria is dynamically regulated by cellular signaling activities [36], and Akt mediates mitochondrial protection in cardiomyocytes [37]. Importantly, Akt suppresses apoptosis signaling independent of cytosolic Akt in cardiac muscle cells [38]. Considering a number of studies addressing the effects of LLI on mitochondria [39], our study may have revealed Akt as an interesting link between LLI and mitochondria.

GSI has been proposed for the treatment of Alzheimer’s disease because its inhibition of GS will decrease the generation of intracellular Aβ, a potential culprit in Alzheimer’s disease [40]. However, GSI treatment will also cause a side effect in healthy cells in Alzheimer’s disease patients since GSI will prevent the normal processing of Notch, whose cleavage by GS at plasma membrane generates ICN, a PTEN suppressor and an Akt enhancer, promoting cell survival [41], [42]. In this study, we observed that GSI down-regulated Aβ and upregulated PTEN, suppressing Akt activation and depressing cell proliferation and cell survival as predicted from previous studies. We also showed that 532 nm LLI was able to decrease PTEN expression of GSI-pretreated cells and to increase Akt expression of those cells while keeping Aβ suppressed. We further demonstrated that the LLI rescued the depression of cell proliferation and even induced further growth. Thus, LLI may be useful to prevent the side effect in the Alzheimer’s disease treatment using GSI. Future studies will examine the combined administration of GSI and 532 nm LLI in animal models of Alzheimer’s disease *in vivo*.
